# DeepO-GlcNAc: a web server for prediction of protein O-GlcNAcylation sites using deep learning combined with attention mechanism

**DOI:** 10.3389/fcell.2024.1456728

**Published:** 2024-10-10

**Authors:** Liyuan Zhang, Tingzhi Deng, Shuijing Pan, Minghui Zhang, Yusen Zhang, Chunhua Yang, Xiaoyong Yang, Geng Tian, Jia Mi

**Affiliations:** ^1^ Shandong Technology Innovation Center of Molecular Targeting and Intelligent Diagnosis and Treatment, Binzhou Medical University, Yantai, Shandong, China; ^2^ National Institute for Data Science in Health and Medicine, Xiamen University, Xiamen, Fujian, China; ^3^ School of Mathematics and Statistics, Shandong University, Weihai, Shandong, China; ^4^ Department of Comparative Medicine, Department of Cellular and Molecular Physiology, Yale University, New Haven, CT, United States

**Keywords:** deep learning, O-GlcNAc, CNN, prediciton, attention

## Abstract

**Introduction:**

Protein O-GlcNAcylation is a dynamic post-translational modification involved in major cellular processes and associated with many human diseases. Bioinformatic prediction of O-GlcNAc sites before experimental validation is a challenge task in O-GlcNAc research. Recent advancements in deep learning algorithms and the availability of O-GlcNAc proteomics data present an opportunity to improve O-GlcNAc site prediction.

**Objectives:**

This study aims to develop a deep learning-based tool to improve O-GlcNAcylation site prediction.

**Methods:**

We construct an annotated unbalanced O-GlcNAcylation data set and propose a new deep learning framework, DeepO-GlcNAc, using Long Short-Term Memory (LSTM), Convolutional Neural Networks (CNN) combined with attention mechanism.

**Results:**

The ablation study confirms that the additional model components in DeepO-GlcNAc, such as attention mechanisms and LSTM, contribute positively to improving prediction performance. Our model demonstrates strong robustness across five cross-species datasets, excluding humans. We also compare our model with three external predictors using an independent dataset. Our results demonstrated that DeepO-GlcNAc outperforms the external predictors, achieving an accuracy of 92%, an average precision of 72%, a MCC of 0.60, and an AUC of 92% in ROC analysis. Moreover, we have implemented DeepO-GlcNAc as a web server to facilitate further investigation and usage by the scientific community.

**Conclusion:**

Our work demonstrates the feasibility of utilizing deep learning for O-GlcNAc site prediction and provides a novel tool for O-GlcNAc investigation.

## Introduction

Protein post-translational modification (PTM) refers to the covalent modification of a protein after synthesized (Conibear, 2020). It plays a crucial role in diversifying protein functions and regulating cellular processes. Among currently known PTMs (Ramazi and Zahiri, 2021), O-linked β-N- acetylglucosaminylation (O-GlcNAcylation) is considered as a critical regulation mechanism (Yang and Qian, 2017). This modification involves the attachment of N-acetylglucosamine (GlcNAc) moieties to serine (S) or threonine (T) residues, a process catalyzed by O-GlcNAc transferase (OGT) and reversed by O-GlcNAcase (OGA) (Yang and Qian, 2017). Among the currently known post-translational modifications (PTMs), O-linked β-N-acetylglucosaminylation (O-GlcNAcylation) is regarded as a critical regulatory mechanism. This modification involves the attachment of N-acetylglucosamine (GlcNAc) moieties to serine (S) or threonine (T) residues, a process catalyzed by O-GlcNAc transferase (OGT) and reversed by O-GlcNAcase (OGA). O-GlcNAcylation plays a vital role as a cellular nutrient and stress sensor, regulating key processes such as signal transduction and cell cycle control (Yang et al., 2020). Its dysregulation has been linked to diseases like cancer, neurodegenerative disorders (Smet-Nocca et al., 2011), and metabolic conditions (Hart et al., 2007; Slawson and Hart, 2011). Identifying O-GlcNAc sites may uncover detailed mechanisms of disease pathology and offer novel therapeutic options. In neurodegenerative diseases, the hyperphosphorylation status of Tau proteins contributes to the neuronal death, and proposed as promising therapeutical targets. The O-GlcNAcylation at residue S400 of the Tau protein may reduce the phosphorylation at S404 which disrupts the GSK3β-mediated sequential phosphorylation process in neuron (Smet-Nocca et al., 2011). Therefore, the elevation of O-GlcNAcylation with O-GlcNAcase inhibitors are proposed as a novel therapy for (Alzheimer’s disease) AD (Arnold et al., 1996; Liu et al., 2004; Morris et al., 2015; Bartolome-Nebreda et al., 2021). In metabolic disorders, the dysregulation of gluconeogenesis is one of the processes that is regulated by Peroxisome proliferator-activated receptor gamma coactivator 1-alpha (PGC-1α). The O-GlcNAcylation at Ser333 of PGC-1α is proved to protect PGC-1α from ubiquitination and further proteasomal degradation, which shedding light on new strategies for diabetes treatment (Ruan et al., 2012). Hence, identifying the specific O-glycosylation sites on proteins of interest is crucial for disease and novel drug investigation.

Bioinformatics-based approach has been proved to be advantageous for PTM site identification, with low cost and high throughout capabilities (Meng et al., 2022; Chen et al., 2019). Predicting potential PTM sites prior to experimental validation has become an essential tool for molecular biologists (Wen et al., 2020; Khan et al., 2021). Early predictors like YinOYang (2002) (Gupta and Brunak, 2002) and O-GlcNAcScan (Wang et al., 2011) used machine learning techniques such as artificial neural networks and support vector machines to improve O-GlcNAcylation site identification. Over time, more advanced models like GlycoMine (Li et al., 2015), further improved prediction performance by coupling the Random Forest (RF) algorithm with effective features selected through information gain (IG) and minimum redundancy maximum relevance (mRMR) (Li et al., 2015). Consideration of protein structural features was also proposed for O-GlcNAc prediction, GlycoMinestruct was constructed for O-GlcNAc prediction based on 29 O-linked glycosylated PDB structures, which corresponded to 47 O-linked glycosylation sites (Li et al., 2016). These predictors have demonstrated the effectiveness of bioinformatics approaches in O-GlcNAc prediction, and some of them have been well adopted by researchers. However, several critical issues persist in O-GlcNAc prediction, such as overall unsatisfactory performance and limited availability of online prediction servers. Therefore, more sophisticated models are needed for improving prediction performance. One potential approach to improve prediction accuracy involves leveraging deep learning-based methods, which have demonstrated success in other PTM predictions (Li et al., 2022a; Wang et al., 2022). Deep learning has presented its remarkable performance in comparison to traditional machine learning methods due to its robustness and generalization. Recently, Hu et al. reported an O-GlcNAc predictor based on connection of a convolutional neural network and bidirectional long short-term memory, indicting the potential of deep learning in O-GlcNAc prediction (Hu et al., 2023). However, the performance is still insufficient, and more algorithms are in need to improve current achievement, such as attention mechanism (Mauri et al., 2021).

In this study, we construct an annotated unbalanced O-GlcNAcylation data set and propose a new deep learning framework, DeepO-GlcNAc, using Long Short-Term Memory (LSTM), Convolutional Neural Networks (CNN) combined with attention mechanism. We have developed a web server for the prediction of protein O-GlcNAcylation sites, making it freely accessible to the public. To evaluate the generalization performance of DeepO-GlcNAc, we incorporate cross-species data from five organisms—mouse, *Drosophila*, *Caenorhabditis-elegans*, *Arabidopsis*, and rat, our model demonstrates strong robustness. Additionally, we conduct ablation experiments to assess our model’s performance. Our model outperforms other architectures such as CNN, CNN-LSTM, CNN-Attention, and CNN-Attention-LSTM, establishing itself as a powerful deep learning-based O-GlcNAc predictor.

**FIGURE 1:**
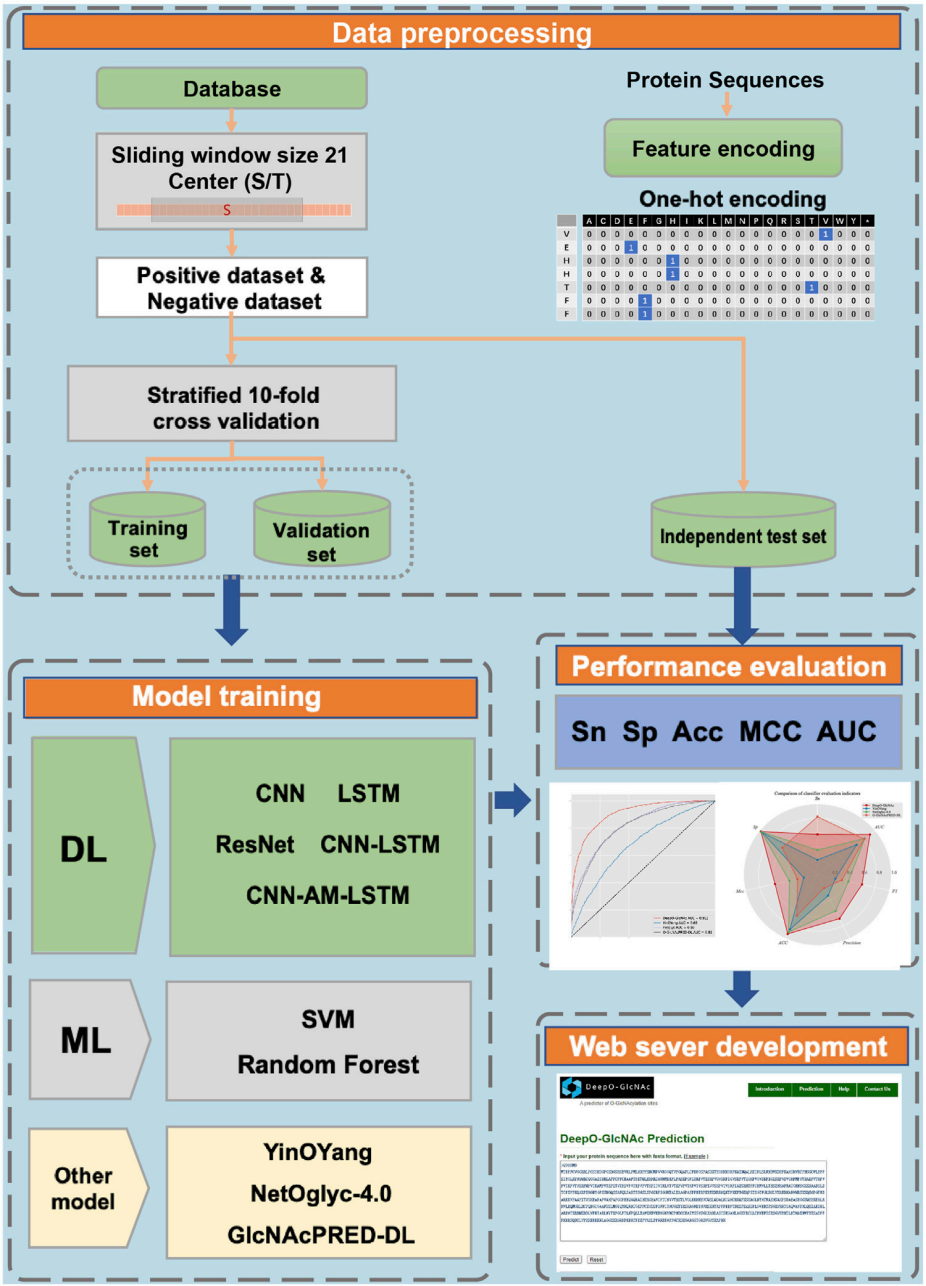
Methodology workflow.

**FIGURE 2:**
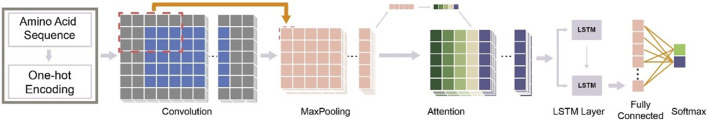
Framework of the fusion of CNN-Attention-LSTM (DeepO-GlcNAc). The amino acid sequences are encoded using one-hot encoding, and passed through a series of layers including Convolution, MaxPooling, Attention, LSTM, and Fully connected layers to construct the framework, then activated by Softmax.

A workflow on ensemble DeepO-GlcNAc is showed in Figure 1. A fixed dataset is retained for testing, with the remaining data as training set. PTM data at the protein level are mapped to core sequences using the slide window of 21 size strategy; One-hot encoding was used to digitize discrete features. Each individual model is trained on the processed data under the label setting; each model is subsequently evaluated in the evaluation steps. An online service of DeepO-GlcNAc was constructed. The framework of CNN-Attention-LSTM model is showed in Figure 2.

## Materials and methods

### Data collection and preparation

We downloaded 4,577 reviewed O-glycosylated protein sequences in dbPTM database (Li et al., 2022b). The obtained 16,691 O-GlcNAcylation sites were experimentally validated. Considering the sequence similarity used in experiments in O-GlcNAcylation site-specific modification assays, we used the CD-HIT tool to remove protein sequences with greater than 30% homology (Li and Godzik, 2006). As the O-glycosylated sites occur in serine or threonine (S/T), we took S or T as the center and intercepted peptide fragments of length 21. Finally, we obtained protein sequences containing a total of 23,252 S/T sites. They can be represented in the following scheme:

$$P\left(0\right)=N_{-10}N_{-9}\ldots N_{-2}N_{-1\;}O{\;N}_{+1}N_{+2}\ldots N_{+9}N_{+10}$$

Where the center O denotes serine (S) or threonine (T). If there are fewer than 21 amino acids, we extended these sequences as virtual amino acids with non-existent residual “*” to ensure that the window length of each sequence was fixed at 21. The peptides fragments can be further divided into two classes:

$$P\left(O\right)\in \left\{\begin{array}{c} P^{+}\left(O\right),\text{if the center is an }O‐\text{glycoslated site},\\ P^{-}\left(O\right),\text{otherwise} \end{array}\right\}$$

where $P^{+}\left(O\right)$ is an experimentally verified O-glycosylated site, i.e., 2,696 positive samples; $P^{-}\left(O\right)$ a non-O-glycosylated site, i.e., 20,556 negative samples.

A total of 23,252 potential sites (Serine and Threonine) are included in the dataset. Among them, 2,696 sites are validated O-GlcNAc sites, and 20,556 sites are considered as negative samples. The dataset was randomly divided into two parts, one part includes 80% of the data as training set and the other with 20% data was used as an independent testing set. A peptide similarity check was performed between two parts with CD-HIT to ensure the testing dataset is independent of training dataset (threshold = 40%) (Li and Godzik, 2006).

To demonstrate the generalization performance of our model, DeepO-GlcNAc was tested on five cross-species benchmark data sets including mouse, *Drosophila*, *Caenorhabditis-elegans*, *Arabidopsis and rats*, the O-GlcNAcylation information in these species were obtained from this website: https://oglcnac.org/atlas/download/. The statistical information on the data is listed in Table 2.

### One-hot encoding

The dataset was encoded with One-hot encoding approach, which is a common and popular feature extraction technique that can generate a numerical feature vector from a protein sequence (Meng et al., 2022). According to this method, one amino acid is denoted as a feature vector of 21-dimension such as amino acid alanine (A) is presented as “100000000000000000000” and the dummy amino acid “*” is presented as “000000000000000000001”. Therefore, an L∗21-dimensional feature vector can be obtained for a protein fragment of length L. In this study, we used window size 21 to generate peptide samples and got a 441 (21 × 21) dimensional feature vector to encode a peptide fragment.

$$B_{i}=\left(b_{n1},b_{n2},b_{n3},b_{n4},\ldots ,b_{n21}\right)$$

$$b\in \left\{\begin{array}{c} \begin{array}{c} \begin{array}{c} \begin{array}{c} A:100000000000000000000\\ C:010000000000000000000 \end{array}\\ \ldots \end{array}\\ Y:000000000000000000010 \end{array}\\ {}*:000000000000000000001 \end{array}\right\}$$

$$n\in \left\{A,C,\ldots ,Y,*\right\}$$

We used a weighted cross-entropy loss function (BCE_Loss), which assigns greater importance to positive samples ensuring that the model does not become biased towards predicting the negative class. The “pos_weight” parameter was opted for 4.

$$\text{BCE}\_\text{Loss}=-\frac{1}{N}\sum_{i=1}^{N}\left[w\cdot y_{i}\cdot \log\left(\sigma \left(x_{i}\right)\right)+\left(1-y_{i}\right)\cdot \log\left(1-\sigma \left(x_{i}\right)\right)\right.$$

### Convolutional neural networks

A convolutional neural network (CNN) architecture was employed for feature extraction and presentation. Convolutional Neural Networks are originally proposed by Fukushima (1980) as noncognition model, which is one of the earliest algorithms in the field of deep learning. This network mainly consists of four layers of operations (Kim, 2014): convolutional layer, pooling layer, fully connected layer, and output layer. The convolution operation is represented mathematically as shown below:

$${\left(I⊗K\right)}_{ij}=\sum_{m=0}^{k_{1}-1}\sum_{n=0}^{k_{s}-1}I\left(i+m,j+m\right)\cdot K\left(m,n\right)$$

Where I is the feature matrix, K is the convolution kernel, i and j represent the i−th and j−th rows and columns of the feature matrix, and m and n represents the m−th and n−th rows and columns of the convolution kernel. Maximum pooling retains the maximum value of each feature, The pooling layer is used to reduce the dimensionality of data, select and filter the features learned, to reduce the complexity of the model and avoid overfitting (Kim, 2014).

Specifically, the CNN model consisted of two convolutional layers (Conv1 and Conv2) followed by rectified linear unit (ReLU) activation functions and max-pooling layers. The first convolutional layer (Conv1) had 10 output channels and a kernel size of 5 × 5, while the second convolutional layer (Conv2) had 20 output channels and a kernel size of 3 × 3. The stride for both convolutional layers was set to 1. The ReLU activation function was applied after each convolutional to introduce non-linearity into the model.

### Long short-term memory

Long Short-Term Memory (LSTM) is a type of recursive neural network extension model proposed by Hochreiter and Schmidhuber (1997). The main advantage of LSTM lies in its internal mechanism of gates that control information flow. With the addition of special “gate” structures, LSTM can handle the problem of long-term memory. The LSTM layer exhibits a hidden state dimensionality of 512. Additionally, two fully connected (dense) layers (FC1 and FC2) followed by the LSTM layer. The first fully connected layer manifests an output dimensionality of 288, serving as an intermediary transformation stage between the LSTM layer and subsequent layers. This dimensionality is selected based on considerations of feature representation and model complexity. The second fully connected layer exhibits an output dimensionality of 2, aligning with the binary classification nature of the task. The ReLU activation function was applied after the first fully connected layer to introduce non-linearity into the model. After the output layer, the log_SoftMax function was employed to compute the logarithm of the softmax probabilities, facilitating model predictions for the binary classification task.

### Attention mechanism

Attention Mechanism is widely applied in various fields such as image and natural language processing, due to its ability to achieve fast parallel computations through matrix operations (Ning and Li, 2022). It calculates the attention distribution on input features and outputs the weighted features based on the attention distribution. Therefore combination of Attention Mechanism may benefit for independent CNN or LSTM network models. The SE block (Hu et al., 2017) is adopted as the core structure of attention in this paper, in order to obtain the importance of each feature channel and the interdependence between feature channels. Weight values are assigned to each feature channel to allow the neural network to focus on these feature channels. For an input of feature channel number C, the weighted feature channels with number C are calculated and then weighted based on the following three operations.

The Squeeze operation uses global average pooling for each channel. It represents the global distribution of responses on feature channels and allows layers near the input to obtain a global receptive field.

$$z_{c}=F_{sq}\left(U_{c}\right)=\frac{1}{H\times W}\sum_{i=1}^{H}\sum_{j=1}^{W}u_{c}\left(i,j\right)$$

The Excitation operation, which is a mechanism similar to the gate in recurrent neural networks, generates weights for each feature channel through the parameter w. The Scale operation considers the weights output by Excitation to represent the importance of each feature channel after feature selection, and then scales the original feature channel through multiplication to complete the re-scaling of the original feature along the channel dimension.

$$\tilde{X_{c}}=F_{scale}\left(U_{c},s_{c}\right)=s_{c}\cdot U_{c}$$

Specifically, two SE blocks were employed after first and second convolutional layer, for the two SE modules, input channels are 10 and 20, corresponding to the output channels of the first and the second convolutional layer.

### Model evaluation

For deep learning model training, ten-fold cross-validation is performed by dividing the dataset into 10 subsets and using 9 of them as training sets and 1 as test set in turn. Each subset is validated once in the ten-fold cross-validation process (Supplementary Figure S1, Supplementary Table S1). The accuracy of each validation is recorded and the model with the highest accuracy is considered as the optimal model. The independent test set is further used to evaluate the model and compare it with the other tools. Several evaluation metrics are employed in this work, including *Accuracy* (*ACC*), *Matthew*’*s correlation coefficient* (*MCC*), *Sensitivity* (*Sn*), *Specificity* (*Sp*), *Precision*, and *F1*-score which are illustrated as

$$Accuracy=\frac{TP+TN}{TP+FP+TN+FN}$$

$$MCC=\frac{TP\times TN-FP\times FN}{\sqrt{\left(TP+FP\right)\left(TP+FN\right)\left(TN+FP\right)\left(TN+FN\right)}}$$

$$Sensitivity=\frac{TP}{TP+FN}$$

$$Specificity=\frac{TN}{TN+FP}$$

$$Precision=\frac{TP}{TP+FP}$$

$$F1=\frac{2\times recall\times precision}{recall+precision}$$

where FP FN TP and TN represent the number of false positives, false negatives, true positives and true negatives, respectively. In addition, we use the area under the ROC (AUC) to measure the classifier’s ability by plotting the true positive rate (TPR) against the false positive rate (FPR).

## Results

### Motif conservation analysis of O-GlcNAc sites in human proteins

**FIGURE 3:**
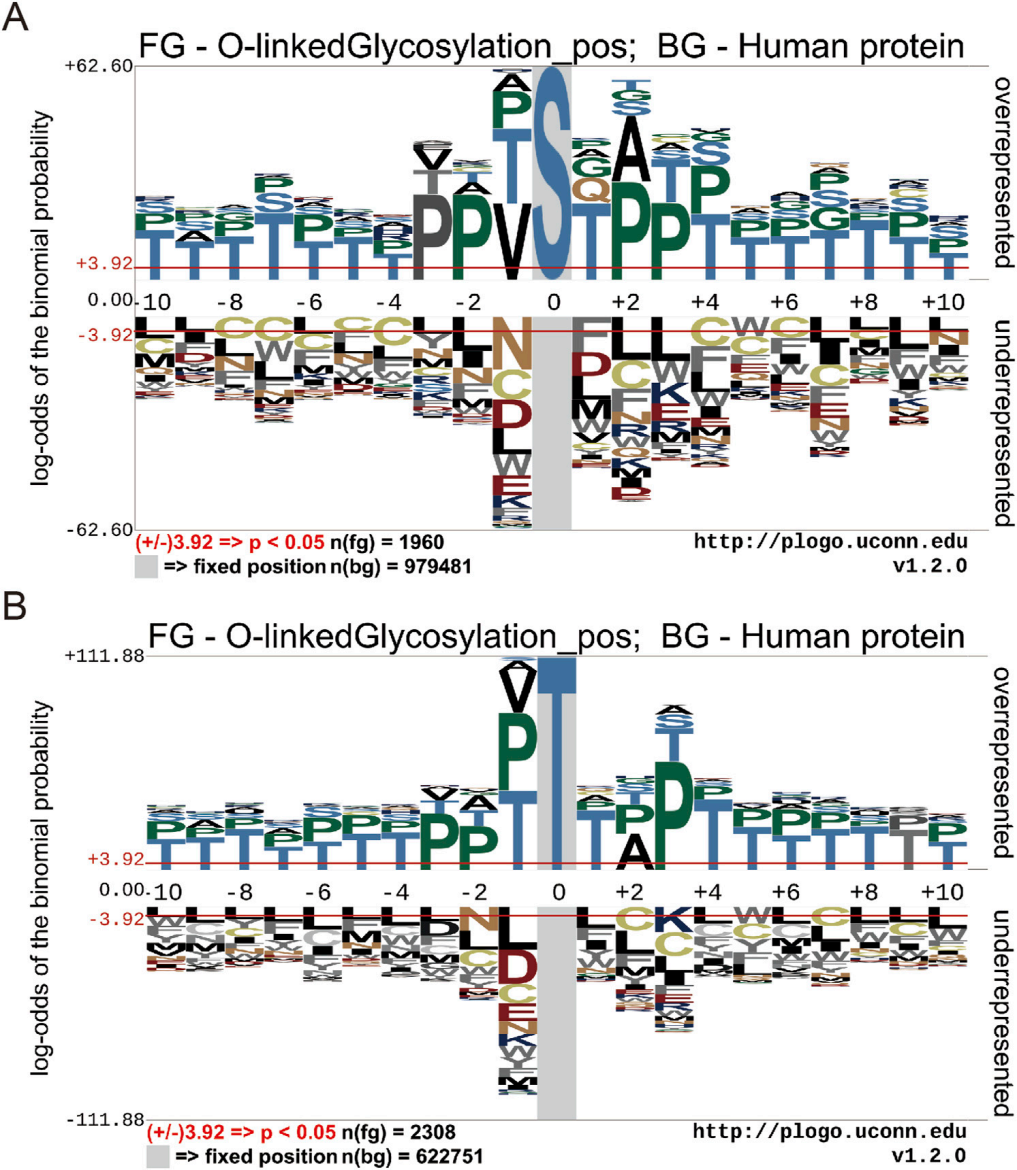
Motif conservation analysis of O-GlcNAc sites. **(A)** Motif conservation analysis of O-GlcNAc sites for Threonine (T) **(B)** Motif conservation analysis of O-GlcNAc sites for serine (S). The sequence logos were generated with pLogo with scaled better data visualization. The default values ± 3.92 (*P* < 0.05) were used as the thresholds for significantly overrepresented and underrepresented amino acids, respectively. The red horizontal lines on the sequence logos denote the *P* < 0.05 threshold.

To illustrate the different distribution and preference of flanking residues surrounding O-GlcNAc sites on human proteins, we used the Probability Logo Generator (pLogo) algorithm. This allowed us to compare the amino acid sequences surrounding observed O-GlcNAc sites with sequences from non-O-GlcNAc sites, utilizing our dataset (Figure 3). Currently, there is no confirmed conservation motif for O-GlcNAc. Our analysis revealed a predominant presence of Threonine (T) and Proline (P) residues in the vicinity of O-GlcNAc sites, whereas Leucine (L) and Cysteine (C) residues were observed around non-O-GlcNAc sites.

### Ablation studies on independent test

**FIGURE 4:**
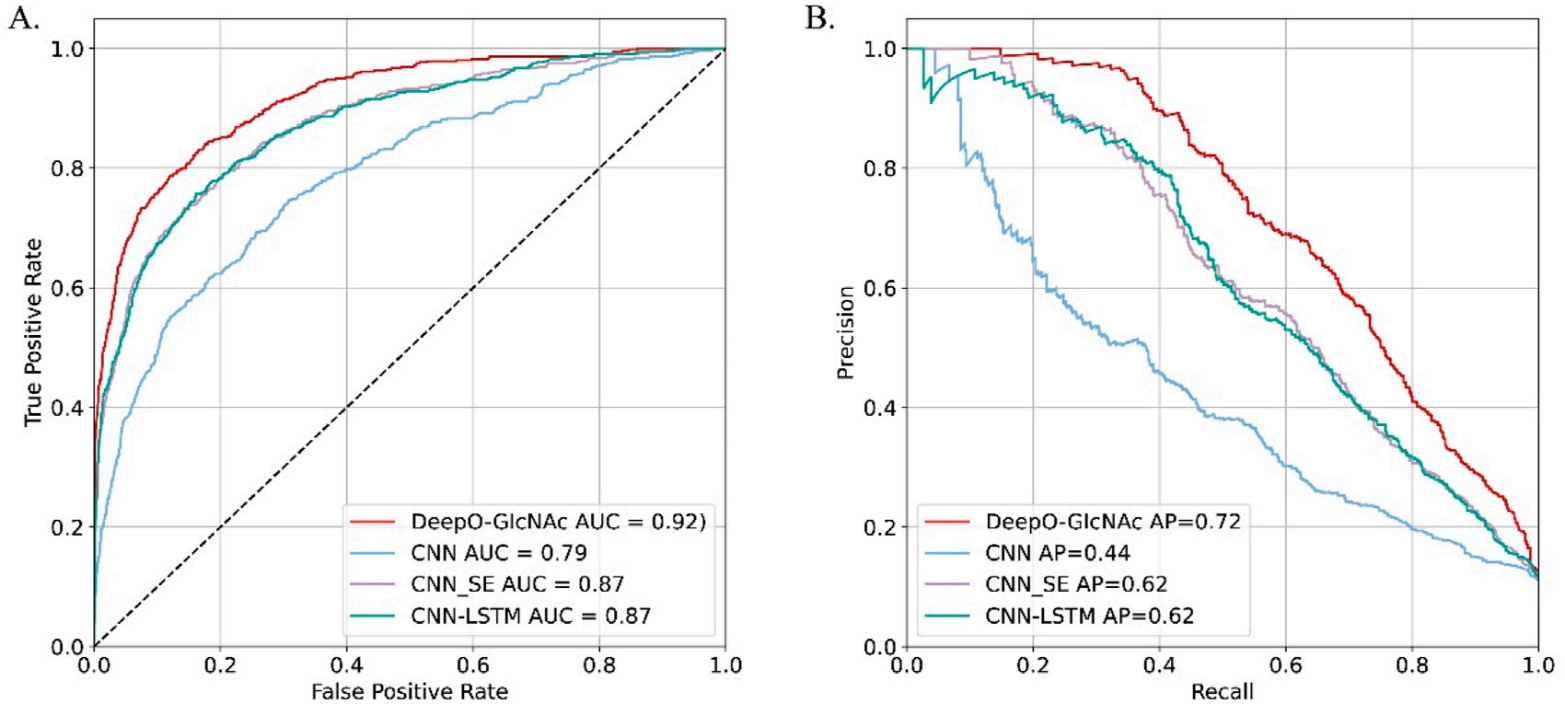
Ablation experiments on DeepO-GlcNAc. **(A)** ROC Curves for O-GlcNAc site prediction models. The ROC curves illustrate the performance of various computational models in predicting O-GlcNAcylation sites on the independent dataset including DeepO-GlcNAc, CNN, CNN_SE, CNN_LSTM. **(B)** Precision-recall curves for O-GlcNAc site prediction models. Precision-recall curves assess the precision against recall for the O-GlcNAcylation site prediction models including DeepO-GlcNAc, CNN, CNN_SE, CNN_LSTM.

To evaluate the impact of different model components on performance of DeepO-GlcNAc. We conducted ablation experiments using an independent dataset. The Area Under the Curve (AUC) values of ROC curves indicating that the DeepO-GlcNAc model (AUC = 0.92) outperforms CNN (AUC = 0.79), CNN-SE (AUC = 0.87), and CNN-LSTM (AUC = 0.87) models in terms of true positive rate versus false positive rate (Figure 4A). In Precision-Recall curves, the Average Precision (AP) of DeepO-GlcNAc (AP = 0.72) exceeds that of the CNN (AP = 0.44), CNN-SE (AP = 0.62), and CNN-LSTM (AP = 0.62) models (Figure 4B). These results highlight the superior performance of DeepO-GlcNAc, especially in reducing false positives and maintaining higher precision across various recall levels. Compared to CNN, CNN_SE, and CNN_LSTM, DeepO-GlcNAc demonstrated the highest sensitivity (Sn = 0.68), Matthews Correlation Coefficient (MCC = 0.60), accuracy (Acc = 0.92), and F1 score (0.65). Meanwhile, CNN_SE achieved the best specificity (Sp = 0.96) and precision (0.62), as shown in Table 1. These results highlight the superior performance of DeepO-GlcNAc and the importance of the SE module in enhancing model performance.

**TABLE 1:** Results of the test data in ablation experiments.

	Sn	Sp	MCC	ACC	Precision	F1	AUC
CNN	0.47	0.91	0.35	0.86	0.39	0.43	0.79
CNN-SE	0.49	**0.96**	0.50	0.91	**0.62**	0.54	0.87
CNN-LSTM	0.54	0.95	0.50	0.90	0.57	0.55	0.87
DeepO-GlcNAc	**0.68**	0.95	**0.60**	**0.92**	0.61	**0.65**	**0.92**

Bold indicates the most significant value among the comparisons of different models.

For each model, the area under the ROC curve and the Precision-Recall curve are reported.

### DeepO-GlcNAc demonstrated varied predictive performance across different species

**FIGURE 5:**
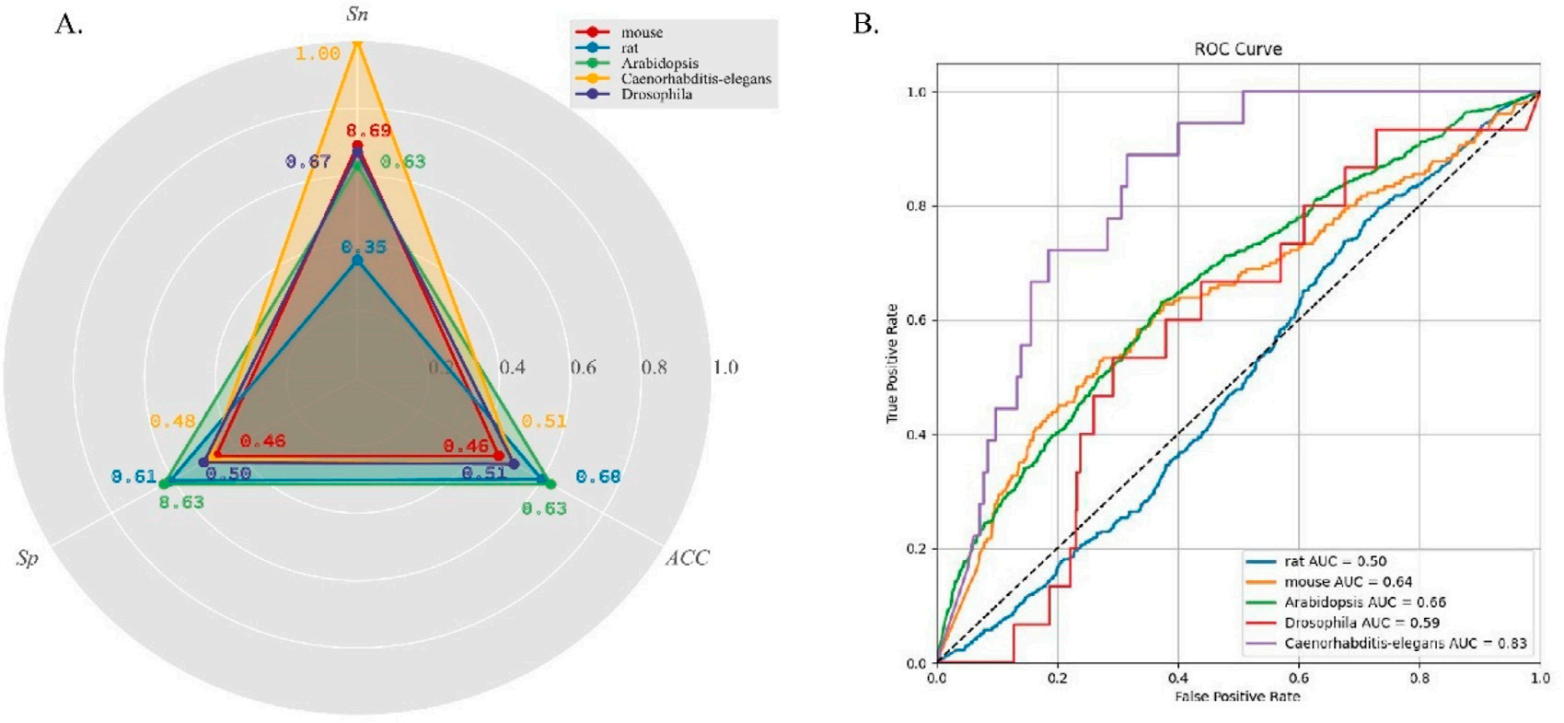
**(A)** Five cross-species datasets testing results on DeepO-GlcNAc. **(B)** Comparison of the ROC curves and AUC values of five cross-species prediction on DeepO-GlcNAc.

To evaluate the generalization performance of DeepO-GlcNAc, we incorporate cross-species data from five organisms—mouse, *Drosophila*, *Caenorhabditis-elegans*, *Arabidopsis*, and rat. The statistical information on the data is listed in Table 2. As can be seen in Figure 5A, accuracy (ACC) values for all species hovered around 0.6, with the highest for *Arabidopsis* at 0.63, while *rat*, *Caenorhabditis-elegans* and *Drosophila* showed slightly lower accuracy values at 0.63 and 0.51. Specificity (Sp) was higher in *Arabidopsis* and *rat*, both achieving values above 0.60. Sensitivity (Sn) was highest in *Caenorhabditis-elegans* and mouse, with the former reaching 1.00. The ROC curves (Figure 5B) further reflect the model’s performance, where *Caenorhabditis-elegans* displays the highest AUC of 0.83, indicating the most reliable predictions, followed by *Arabidopsis* with an AUC of 0.66. Conversely, *rat* had the lowest AUC of 0.50, suggesting limited predictive power for this species.

**TABLE 2:** Statistical information on species apart from human species.

Species	Positive	Negative
mouse	180	11,240
*Drosophila*	101	6,212
*Caenorhabditis-elegans*	86	1,538
*Arabidopsis*	548	24,028
rats	454	10,375

### Performance comparison between DeepO-GlcNac and current predictors

**FIGURE 6:**
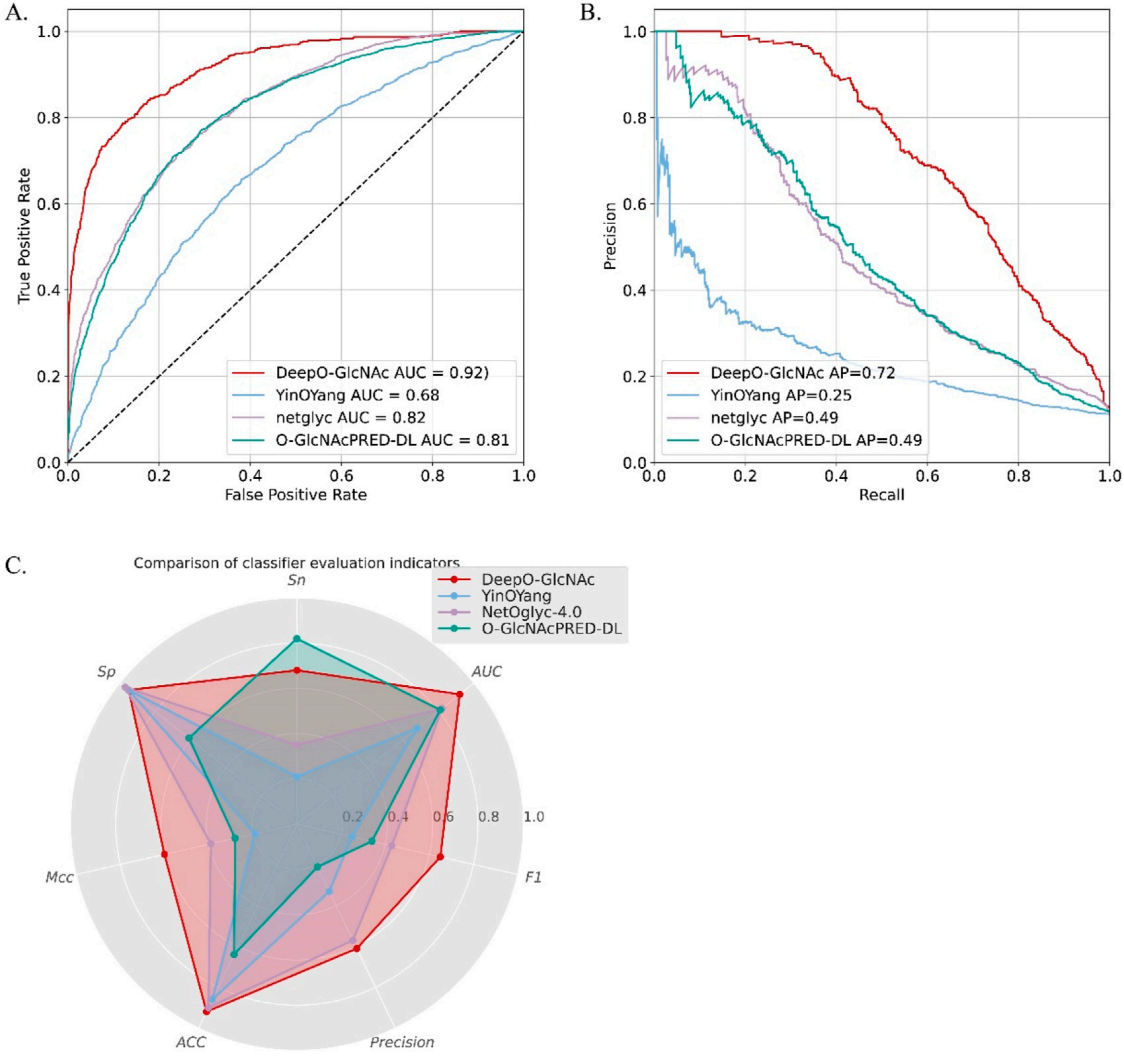
The performance comparison between DeepO-GlcNAc to three currently existing predictors, including YinOYang, NetOglyc-4.0, and GlcNAcPRED-DL. **(A)** The ROCs of different predictors are compared based on the independent test dataset. DeepO-GlcNAc presents the best AUC of 0.92 compared to YinOYang (0.68), NetOglyc-4.0 (0.82) and GlcNAcPRED-DL (0.81). **(B)** The PR plots of different predictors are compared. DeepO-GlcNAc presents the best AP of 0.72 compared to YinOYang (0.25), NetOglyc-4.0 (0.49) and GlcNAcPRED-DL (0.49). **(C)** The Radar plot displayed the metrics of Sp, Sn, AUC, F1, Precision, ACC and MCC. DeepO-GlcNAc, YingOyang, NetOglyc-4.0 and GlcNAcPRED-DL are shown in red, blue, purple, and green respectively. DeepO-GlcNAc ranks first in Precision, AUC, F1, ACC, and MCC, and second in Sn among the predictors.

To demonstrate the predictive capability and robustness of DeepO-GlcNAc, we conducted a performance comparison with other currently available predictors. We compared our model with two available web services and the most-recently released O-GlcNAc prediction tool, including YinOYang, NetOglyc-4.0, and GlcNAcPRED-DL. The independent test dataset was submitted to all the predictors and the results were compared parallelly. DeepO-GlcNAc outperformed all the tools in terms of accuracy (ACC), specificity (Sp), and AUC, Matthew’s correlation coefficient (MCC), F1 score and Precision. It achieves an AUC value of 0.92, which is 24% higher than YinOYang, 10% higher than NetOglyc-4.0% and 11% higher than GlcNAcPRED-DL (Figure 6). Our model has the highest precision of 0.61, ACC of 92%, as well as the highest MCC of 0.60. Metrics related to class balance, such as precision-recall curves and the F1-score, DeepO-GlcNAc performs the best. These results highlight the advantages of DeepO-GlcNAc (Table 3). And we also provide the list of the independent dataset as a supporting material including which specific sites and proteins were successfully identified when using a certain tool for benchmarking (Supplementary Table S1).

**TABLE 3:** Performance comparison of DeepO-GlcNAc with other prediction models.

Tool	Sn	Sp	MCC	ACC	Precision	F1	AUC
DeepO-GlcNAc	0.68	0.95	**0.60**	**0.92**	**0.61**	**0.65**	**0.92**
YinOYang	0.21	0.95	0.19	0.86	0.33	0.25	0.68
NetOglyc-4.0	0.35	0.97	0.39	0.90	0.57	0.43	0.82
GlcNAcPRED-DL	**0.82**	0.61	0.28	0.64	0.21	0.34	0.81

Bold indicates the most significant value among the comparisons of different models.

### Implementation of the DeepO-GlcNAc webserver

To facilitate the usage of DeepO-GlcNAc by other researchers, we have developed a user-friendly web server based on DeepO-GlcNAc. The online service of DeepO-GlcNAc was constructed in an easy-to-use manner using Flask and HTML. The model was deployed in Tencent Cloud, which is equipped with 16 cores, 64 GB memory and a 2 TB hard disk. It was developed using the Windows Sever 2016-Flask-HTML open-source web platform and has been extensively tested on various web browsers including Internet Explorer, Mozilla, Firefox and Google Chrome to provide a robust service. For convenience, the online service of DeepO-GlcNAc was implemented and freely available at http://124.220.189.245:8000/.

Supplementary Figure S2 showcases the user interface of the server, along with an example of prediction output. The server is hosted on the Tencent cloud computing facility. The server utilizes DeepO-GlcNAc to identify O-GlcNAc sites within submitted protein sequences. On the index webpage, users can conveniently submit FASTA formatted protein sequences in the provided textbox. The prediction results include comprehensive information such as the positions of predicted modification sites, corresponding scores, and the overall prediction outcomes. Users also have the option to download the generated prediction results in plain text format for further analysis. In addition, the curated benchmark datasets and the independent test dataset used in our study are available for downloaded from the O-GlcNAc web server (Supplementary Figure S2).

## Discussion

In this work, we built a dataset containing 700 experimentally validated O-glycoproteins from humans. Then, we specifically designed three neural network frameworks—CNN, LSTM, and Attention, respectively—to extract protein sequence features. In our analysis, based on the results of ablation experiments, the combination of CNN, LSTM and Attention presented the best performance. Both CNN and LSTM have been proved efficient in PTM prediction (Naseer et al., 2021). CNN excels at capture spatial patterns inherent in the input data. While LSTM networks are adept at capturing sequential patterns and long-range dependencies. We consider that both local sequence patterns and the temporal of these patterns are crucial for O-GlcNAc modification prediction. Therefore, LSTM complements this by processing the spatial feature maps across spatial dimensions to capture temporal dependencies among features for each sequence. Through this fusion, the model gains a comprehensive understanding of both spatial and sequential characteristics associated with O-GlcNAc modification sites, ultimately enhancing its predictive performance. We also deployed an attention mechanism which introduces an adaptive approach where the importance of each channel is individually assessed based on its context. It has been proved that attention mechanism yield substantial performance enhancements in state-of-the-art CNNs (Hu et al., 2020). In our model, the utilization of attention mechanism improved the model’s AUC value from 0.87 in the CNN-LSTM architecture to 0.92. This suggests that certain amino acid patterns are more critical for O-GlcNAc prediction. Given that the detailed mechanism of protein O-GlcNAcylation is not fully clear, this information could be valuable for further investigation. Moreover, by suppressing redundant or irrelevant feature maps, the attention mechanism enhances the model’s generalization ability and robustness. Consequently, LSTM can focus more on valuable feature information pertinent to the prediction task, thus enhancing the overall performance of the model.

Deep learning-based approaches have been widely applied to various types of PTM prediction, and their advantages have been well demonstrated (Meng et al., 2022; Naseer et al., 2022). However, the application of deep learning in O-GlcNAc prediction has not yet achieved significant success (Mauri et al., 2021). Previous attempts using the CNN for O-GlcNAc prediction did not yield substantial improvements (Hu et al., 2023; Zhu et al., 2022), possibly due to the relatively small dataset sizes and model construction limitations. In our study, we benchmarked five deep learning-based models and demonstrated the potential of the CNN-Attention-LSTM fusion model through both independent testing and cross-validation. This indicates the feasibility of using deep learning in O-GlcNAc prediction and suggests that further optimization of deep learning models could enhance the prediction performance. Based on the test results of incorporating cross-species data from five organisms—mouse, *Drosophila*, *Caenorhabditis-elegans*, *Arabidopsis*, and rat, our model demonstrates strong generalization performance.

Despite the existence of several algorithms for O-GlcNAc prediction, the availability of public online services is still limited. Currently, only a few O-GlcNAc prediction services are accessible, which hampers O-GlcNAc research. In this study, we developed a free online service platform based on the CNN-Attention-LSTM model. Compared to the other existing servers, our server demonstrated improved performance in sensitivity, specificity, and precision. Thus, our sever can be a new helpful tool for O-GlcNAc research.

There are still several limitations in our work. In the study, we used a dataset with 2696 O-GlcNAcylation sites experimentally validated. Due to the nature distribution, it is an imbalanced dataset with more negative peptides than positive ones. Even it may better reflect the actual situation, such imbalance may make deep learning algorithms tend to be biased toward the negative class. This could potentially explain why our model exhibited less sensitivity compared to O-GlcNAcPRED-DL, which utilized a balanced dataset. On the other hand, our predictor and another two predictors with imbalanced dataset presents better specificity compared to O-GlcNAcPRED-DL. Whether and how should we deal with such kind of dataset in PTM prediction need to be further investigated. In addition, we employ sliding window method to construct O-GlcNAc sites, which is commonly in PTM predication. However, this method introduces information redundancy and may lead to inefficient resource utilization. Moreover, it captures local sequence information, and neglects the overall structure within the global sequence. This limitation may be improved by optimizing the length of the window.

In summary, the developed DeepO-GlcNAc predictor achieved remarkable performance in O-GlcNAc site prediction. Its success demonstrates the feasibility of using deep learning for O-GlcNAc prediction, and the online predictor service provides a valuable tool for future research in this field.

## Data Availability

The original contributions presented in the study are included in the article/Supplementary Material, further inquiries can be directed to the corresponding author.
