# Supplementary material for: DeepO-GlcNAc: a web server for prediction of protein O-GlcNAcylation sites using deep learning combined with attention mechanism
Source: Front Cell Dev Biol. 2024 Oct 10;12:1456728. doi: 10.3389/fcell.2024.1456728 (PMC11500328; doi:10.3389/fcell.2024.1456728)
Supplement: Supplementary file 2 [file Image1.pdf]

DeepO-GlcNAc: A Web Server for Prediction of Protein O-GlcNAcylation Sites Using Deep Learning

Running title O-GlcNAcylation site prediction with deep learning

Liyuan Zhang [1#], Tingzhi Deng [1,2#], Shuijing Pan [1], Minghui Zhang [1], Yusen Zhang[3], Chunhua Yang [1], Xiaoyong Yang [4], Geng Tian [1], Jia Mi[1]*

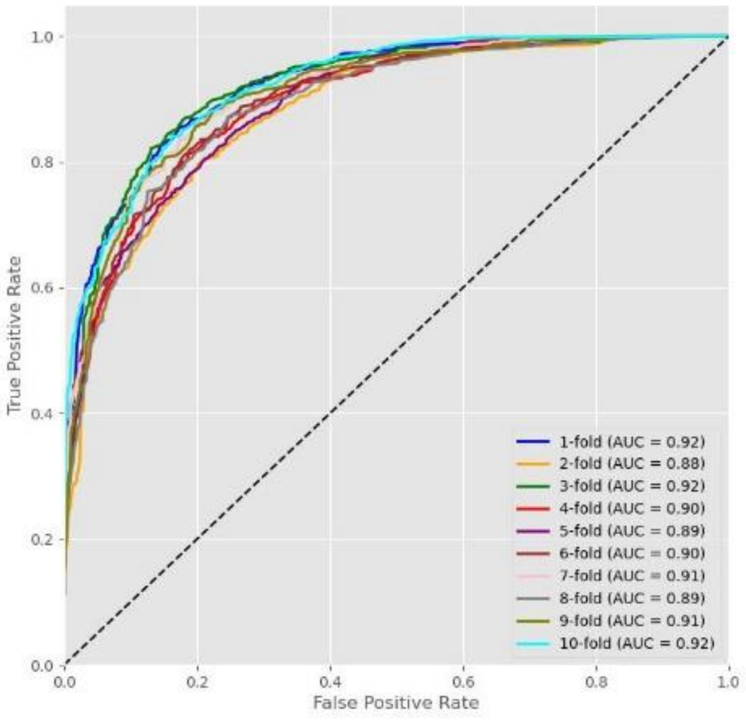

Supplementary Figure S1. ROC plot. Performance of DeepO-GlcNAc 10-fold cross validation on the training dataset. The AUC ranges from 0.88 to 0.92 in the cross validation.

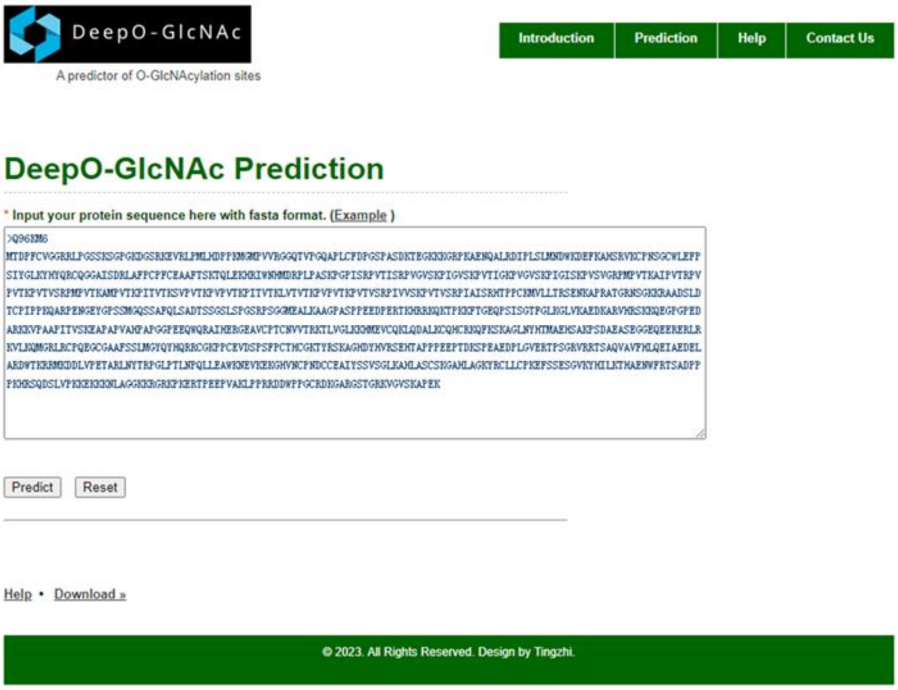

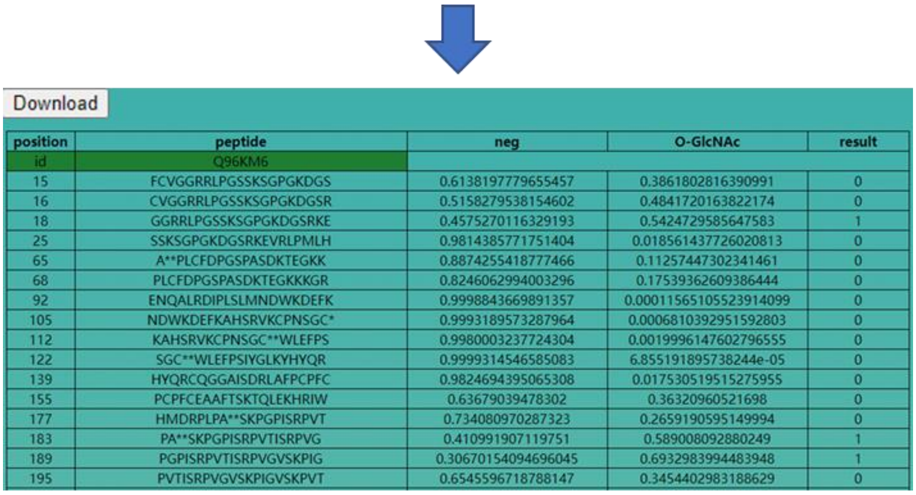

position	peptide	neg	O-GlcNAc	result
id	Q96KM6			
15	FCVGGRRLPGSSKSGPGKDGS	0.6138197779655457	0.3861802816390991	0
16	CVGGRRLPGSSKSGPGKDGSR	0.5158279538154602	0.4841720163822174	0
18	GGRRLPGSSKSGPGKDGSRKE	0.4575270116329193	0.5424729585647583	1
25	SSKSGPGKDGSRKEVRLPMLH	0.9814385771751404	0.018561437726020813	0
65	A**PLCFDPGSPASDKTEGKK	0.8874255418777466	0.11257447302341461	0
68	PLCFDPGSPASDKTEGKKKGR	0.8246062994003296	0.17539362609386444	0
92	ENQALRDIPLSLMNDWKDEFK	0.9998843669891357	0.00011565105523914099	0
105	NDWKDEFKAHSRVKCPNSGC*	0.9993189573287964	0.0006810392951592803	0
112	KAHSRVKCPNSGC**WLEFPS	0.9980003237724304	0.0019996147602796555	0
122	SGC**WLEFPSIYGLKYHYQR	0.9999314546585083	6.855191895738244e-05	0
139	HYQRCQGGAISDRLAFPCPFC	0.9824694395065308	0.017530519515275955	0
155	PCPFCEAAFTSKTQLEKHRIW	0.63679039478302	0.36320960521698	0
177	HMDRPLPA**SKPGPISRPVT	0.734080970287323	0.2659190595149994	0
183	PA**SKPGPISRPVTISRPVG	0.410991907119751	0.589008092880249	1
189	PGPISRPVTISRPVGVSKPIG	0.30670154094696045	0.6932983994483948	1
195	PVTISRPVGVSKPIGVSKPVT	0.6545596718788147	0.3454402983188629	0

Supplementary Figure S2. Screenshots of the DeepO-GlcNAc web server for prediction O-GlcNAc sites. (A) The interface of the DeepO-GlcNAc web server, (B) The prediction output example of DeepO-GlcNAc. The results includes the location of potential O-GlcNAc sites, the peptides around the sites, predicted negative and positive scores, and the prediction results based on the threshold of 0.5. 0 represents negative and 1 represents positive.
